# Body Composition in Children: What Does It Tell Us So Far?

**DOI:** 10.3390/children9081199

**Published:** 2022-08-10

**Authors:** Odysseas Androutsos, Antonis Zampelas

**Affiliations:** 1 Laboratory of Clinical Nutrition and Dietetics, Department of Nutrition and Dietetics, University of Thessaly, Thessaly, 42132 Trikala, Greece; 2 Laboratory of Dietetics and Quality of Life, Department of Food Science and Human Nutrition, Agricultural University of Athens, 11588 Athens, Greece

The Special Issue “Body Composition in Children” of the journal “CHILDREN” aimed to publish both original and review articles focusing on the prevalence and determinants of obesity across childhood, the role of body composition in children’s health, new approaches to assessing body composition, and interventions aiming to improve body composition in children and adolescents.

Body composition plays an important role in children’s health and influences their energy requirements. It is determined by several factors, including genetic predisposition, age, gender, ethnicity, perinatal factors, energy balance (nutrition, physical activity, and sedentary behavior), and health status, while it can be assessed using various complex techniques (e.g., the four-compartment model) or more surrogate methods (anthropometry, bioelectrical impedance analysis (BIA), etc.). Developing new tools, standardizing the assessment methods, and evaluating the validity and applicability of existing or novel methods of assessing body composition in pediatric populations would help to optimize nutritional assessment and enable the scientific community to overcome important barriers related to its applicability in public health actions and in clinical practice.

Furthermore, understanding the underlying mechanisms linking body composition and health is essential. Studies exploring the pathways through which adiposity induces changes in health indices are required in order to tackle the effects of obesity on children’s metabolic profile and quality of life, and on the development of chronic diseases across the lifespan. On the other hand, exploring the effectiveness of lifestyle interventions in improving children’s body composition is essential for the prevention of obesity.

To shed further light on this field, fifteen articles following different designs and methodologies were included in the present Special Issue, and their major findings are given in Table 1.

The cross-sectional study by Markovic et al. reported prospective changes in the prevalence of obesity within a national representative sample of primary schoolchildren (*n* = 6105) in Serbia [1]. Data were collected in 2015 and 2019, and the children were categorized according to the International Obesity Task Force (IOTF) and the WHO criteria. The results of this study showed that the prevalence of overweight/obesity increased by 4.1% based on the WHO criteria, or by 7.2% based on the IOTF criteria, indicating that the monitoring and surveillance of pediatric malnutrition should be included in Serbia’s public health agenda.

Georgiou et al. conducted a cross-sectional study to record the nutritional habits and to explore the level of adherence to the Greek food-based guidelines among children and adolescents classed as overweight or obese (*n* = 1467, 2–18 years old) [2]. According to the findings of this study, the consumption of various core food groups (dairy products, fruit, vegetables, legumes, and fish) was lower than the national recommendations, while the consumption of meat/poultry exceeded them. Moreover, a high number of the participants were found to consume unhealthy foods and beverages (e.g., regular soft drinks, sweets, salty snacks, etc.) regularly. The findings of this study indicate that a large proportion of overweight/obese children and adolescents in Greece do not adhere to the national food-based recommendations; therefore, public health initiatives must be carried out to improve their dietary habits and prevent further increases in their body weights.

The studies by Magriplis et al., Karatzi et al., and Ferrer-Santos et al. aimed to provide more evidence regarding the determinants of obesity in children and adolescents [3,4,5]. More specifically, Magriplis et al. conducted a cross-sectional study to record sugar intake in Greek children and adolescents and to explore its association with overweight/obesity [3]. The study sample included 1165 children/adolescents aged ≥ 2–18 years, who participated in the Hellenic National Nutrition and Health Survey (HNNHS). According to the results of this study, a large proportion of the participants (18.7% of children and 24.5% of adolescents) exceeded the recommendation of 10% of total energy intake from added sugars, which may be attributed to their high consumption of foods rich in sugar, such as sweets and processed/refined grains and cereals in the case of children, or sugar-sweetened beverages in the case of adolescents. Furthermore, individuals with an intake of ≥10% of their total energy from added sugars were more likely to be overweight/obese compared to their peers with an intake of <10%, even after adjusting for the intake of other foods or macronutrients. In their systematic review, Karatzi and her colleagues explored the impact of the COVID-19 pandemic on children’s and adolescents’ lifestyles and cardiovascular risk factors [4]. The electronic search resulted in 15 studies, which met the eligibility criteria set by the authors. It was revealed that the prolonged measures taken to fight against the COVID-19 pandemic had negative effects on children’s and adolescents’ dietary and lifestyle behaviors (e.g., an increase in the consumption of fats, fast-foods, processed foods, sweet and salty snacks, and sugar-sweetened beverages, as well as an increase in screen-time and a decrease in physical activity). However, on the other hand, a few studies also reported some improvements (e.g., an increase in the frequency of breakfast consumption and in fruit and vegetable intake, and a decrease in soft drinks consumption). Such differences could be attributed to the different lockdown periods addressed by each study and the diversity of the measures applied across different countries, as well as the participants’ adherence to these measures. This study also reported that the COVID-19 period negatively affected children’s and adolescents’ weight status and body composition. On the other hand, the more significant presence of parents during the home confinement lead to greater support for type-1 diabetic children, who ultimately achieved better glycemic control. The study by Ferrer-Santos et al. aimed to assess the association between moderate-to-vigorous physical activity (MVPA) and body composition in 308 schoolchildren (aged 7 years old) in Aragon, Spain [5]. Gold standard techniques were used to assess both the participants’ body composition (i.e., dual-energy X-ray absorptiometry) and their physical activity (i.e., accelerometers) and included several potential covariates (e.g., lifestyle, body weight gain during the first year of life, parental BMI, smoking status, etc.). Based on the results of this study, MVPA was inversely associated with subtotal and abdominal fat, indicating that the level and intensity of physical activity constitute important determinants of childhood obesity.

Rusek et al. conducted a study based on 464 schoolchildren aged 6–16 years in Poland to examine the alterations in their body composition and posture during puberty growth [6]. The results of this study showed that children with higher BMIs tended to have higher percentages of body fat, while younger children had lower percentages of body fat. Moreover, girls experienced a greater increase in adipose tissue (both in the total and segmental body composition analyses), and boys in lean mass, muscle mass, and total body water. Pubertal status also appeared to influence the children’s posture. The findings of this study indicate the necessity of screening children periodically to assess their body composition and posture, and of early intervening, wherever needed, to prevent any adverse consequences of these factors on their health status.

The studies by Hsu CU et al., Tengku et al., Jeong et al., and Hsu CN et al. examined the effects of body composition on health indices and quality of life among healthy or ill children [7,8,9,10]. More specifically, Hsu CU et al. aimed to shed more light on the impact of body composition on children’s level of physical fitness [7]. In total, 360 children were recruited from elementary schools in Northern Taiwan. Their body composition was assessed using BIA and their fitness level using different tests and outcomes (800 m run to assess cardiorespiratory fitness; sit-and-reach exercise to assess flexibility; 1 min sit-ups to assess speed/agility; and long-jump to assess lower body power). The findings of this study showed that the % of body fat was independently associated with cardiorespiratory fitness, and the BMI z-score was associated with flexibility. Based on these observations, the authors concluded that weight management and physical fitness should be addressed simultaneously in pediatric populations. Similarly, Tengku et al. conducted a prospective study focusing on the association between the oral disease burden and oral health related quality of life in adolescents, based on their weight status [8]. In total, 195 adolescents classed as overweight/obese and 202 adolescents classed as a normal weight, who were matched by age (14 years at the baseline) and gender, were followed up for two years (2015–2017) in Malaysia. No significant differences were observed between the two groups regarding the burden of oral diseases and indices of oral health related quality of life, thus indicating that obesity in adolescence does not appear to negatively affect these health indices. Jeong et al. compared hematological indices among children and adolescents of different weight categories [9]. The subjects participated in the Korea National Health and Nutrition Examination Survey (KNHANES) in 2007–2018, and, for the purposes of this study, 7997 children/adolescents (4259 boys) were included. The obese participants were found to have higher levels of white blood cells, red blood cells, and platelets compared to their normal-weight peers. Moreover, positive associations were observed between BMI standard deviation scores and white blood cells, red blood cells, hemoglobin, hematocrit, and platelets. As shown in this study, body composition influences hematological parameters and, therefore, children and adolescents with obesity should be periodically screened to assess their levels of these indices. Hsu CN et al. conducted a prospective cohort study based on 63 children and adolescents (8–18 years old) from Taiwan with chronic kidney disease to examine the association of body composition with cardiovascular risk factors [10]. The fat mass index (FMI) z-score was found to be associated with ambulatory blood pressure monitoring, indicating that body composition may influence the levels of certain cardiovascular risk factors and could be considered as a new method for identifying children and adolescents with early-stage chronic kidney disease, who are at a high risk of developing cardiovascular disease.

The studies of Ofenheimer et al., Valencia-Sosa et al., and Thajer et al. focused on the use and utility of certain body composition indicators in healthy or ill children [11,12,13]. In particular, Ofenheimer et al. explored serum lipid profiles in relation to body composition in a large cohort of 1394 children and adolescents (6–<18 years old), who participated in the LEAD study in 2011–2019 [11]. The body composition analysis was performed with dual X-ray absorptiometry (DXA), and the appendicular lean mass (ALMI) and fat mass (FMI) indices were calculated. It was observed that children with high ALMI and high FMI had abnormal levels of certain blood indices, including high levels of triglycerides and LDL cholesterol and low levels of HDL cholesterol. The results of this study also suggest that BMI does not adequately reflect body composition/adiposity. Consequently, more precise methods should be in use to assess body composition and to identify the children who are at a higher risk of developing cardiovascular disease.

Valencia-Sosa et al. provided reference values for neck circumference among Mexican children, a method which was introduced about a decade ago as a new technique for screening for obesity, especially central obesity, and cardiometabolic risk indices in pediatric populations [12]. A number of 1059 schoolchildren aged 6–11 years old with normal weight participated in this study. Overall, the authors recorded an age-dependent increase in the values of neck circumference, as well as a gender-specific difference, since boys had higher values compared to girls. Considering that neck circumference is a valid, simple, low-cost, and easy-to-apply method of screening for obesity and cardiometabolic risk factors, the publication of the percentile reference values for Mexican children is expected to contribute to the early identification of high-risk children and improve public health in Mexico.

Thajer et al. compared different methods of BIA in 123 children and adolescents (6–18 years old) with obesity in Austria [13]. More specifically, two BIA devices, namely TANITA and BIACORPUS, were used to assess body fat in all the study participants, while gold standard techniques (i.e., dual X-ray absorptiometry and air displacement plethysmography) were also applied in a subsample of volunteers. The agreement between the measurements taken by the two BIA methods and the agreement between each BIA method and each gold-standard method were tested using Bland–Altman analyses. The study showed that the BIA methods produced different results in the same individuals, with TANITA overestimating the % of body fat and the fat mass compared to BIACORPUS and underestimating the fat-free mass. Gender-specific differences were also identified. These findings suggest that both BIA devices can be used in clinical practice and research. However, it is important to use the same type of device in order to be able to compare a series of measurements taken at the individual level for clinical assessment and to compare measurements taken by different individuals in population-based studies.

The last two studies by Bielec et al. and Trajkovic et al. examined the impact of physical activity on children’s and adolescents’ body composition [14,15]. In brief, Bielec et al. compared body composition and anthropometric indices between children 11–12 years old who participated regularly in swimming activities (e.g., football, basketball, athletics, etc.) (*n* = 46) or other sports, including non-swimming activities (*n* = 42) for 5–12 h/week over a period of 12 weeks [14]. It was observed that participants’ body weights and heights increased in both groups after the 12-week period; however, BMI and body fat percentage increased only among the non-swimmer groups. The authors noted, however, that the group of swimmers engaged in higher volumes of training compared to their non-swimmer peers, which may explain the study findings. It was concluded that the engagement for >10 h/week in vigorous exercise, such as swimming, may prevent excess body fat increase in children.

Trajkovic et al. evaluated the effects of an after-school volleyball program on body composition indices among overweight adolescent girls in Serbia [15]. The participants were randomly allocated to the intervention group (*n* = 22) or the control group (*n* = 20) and attended the program for 12 weeks. Both groups followed their standard PE activities, and the intervention group also engaged in small-sided games two times/week. Body composition was analyzed using BIA. The study showed that the after-school volleyball program improved body composition among the intervention group compared to the control group, indicating that it may be a useful strategy for tackling overweight/obesity in adolescent girls.

In conclusion, the studies included in the Special Issue “Body Composition in Children” of the journal “CHILDREN” showed that the prevalence of childhood obesity is increasing in certain areas of Europe. This increase can be attributed, to a large extent, to the adoption of unhealthy dietary and lifestyle behaviors by children and adolescents, which may lead to poor health status and quality of life and pose an economic burden for the public health systems. New methods, such as the neck circumference technique, may be used to identify children/adolescents at a high risk of developing cardiovascular disease and guide them within the health care system so that they can be treated accordingly. In parallel, effective strategies and interventions to promote healthy lifestyles were proposed, and these may be incorporated into future obesity prevention/treatment actions targeting children and adolescents.

## Figures and Tables

**Table 1:** Short description and main findings of the studies published in the Special Issue “Body Composition in Children” of the journal “CHILDREN”.

*n*/*n*	Authors	Title	Study Population	Study Design	Type of Intervention/Exposure/Data Collection Method	Main Findings/Outcomes
1	Markovic et al. [1]	Childhood Obesity in Serbia on the Rise	Primary school children aged 6.00 to 8.99 years in 2015 and 6.00 to 9.99 in 2019. In both rounds, nationally representative samples were selected through cluster sampling, with the primary school as the primary sampling unit. Schools were selected randomly from the list of public primary schools provided by the Serbian Ministry of Education. Since less than 1% of the target children were enrolled in private or special schools, these schools were excluded from the sampling frame. Stratification by the region, district, and level of urbanization was applied. The minimal planned sample size consisted of 2400 children per round, as recommended by the WHO European Office.	Cross-sectional study (2015 & 2019)	WHO COSI-design anthropometric measurements (body weight and height). Calculation of BMI and conversion to BMI categories (IOTF criteria and WHO criteria).	Prevalence of overweight/obesity in 7–9-year-old children increased by 4.1% (2015: 30.7%, 2019: 34.8%, *p* < 0.05) based on the WHO criteria, or 7.2% (2015: 22.8%, 2019: 30.0%, *p* < 0.05) based on the IOTF criteria.
2	Georgiou et al. [2]	Do Children and Adolescents with Overweight or Obesity Adhere to the National Food-Based Dietary Guidelines in Greece?	Children and adolescents aged 2–18 years (*n* = 1467) attending the out-patient clinic for the prevention and management of overweight and obesity in childhood and adolescence were recruited between October 2014 and March 2017.	Cross-sectional study	Anthropometric measurements (body weight and height). Calculation of BMI and conversion to BMI categories (IOTF criteria). FFQ & Questionnaire (SES, medical issues)	The consumption of dairy products, fruits, vegetables, legumes, and fish by children/adolescents with overweight or obesity was lower than the national recommendations (ranging from a minimum of 39.5% for fish to a maximum of 75.5% for cereals/potatoes/rice). The consumption of meat/poultry was found to exceed the national recommendation (estimated coverage of 131.3%). A large proportion of participants regularly consumed various unhealthy foods/beverages.
3	Magriplis et al. [3]	Dietary Sugar Intake and Its Association with Obesity in Children and Adolescents	Hellenic National Nutrition and Health Survey (2013–2015). Multi-stage stratified design aimed at achieving representativeness in six groups (0–19 years, 20–65 years, and 65+, in males and females), across four geographical regions of Greece. For this study, data from 1165 children & adolescents aged ≥2–18 years (66.8% males) were used (*n* = 781; aged 2–11 years) and adolescents (*n* = 384; aged 12–18 years). Those who reported energy intakes of >6000 kcal/day were excluded to avoid extreme over-reporting.	Cross-sectional study	Anthropometric measurements (body weight and height). Calculation of BMI and conversion to BMI categories (IOTF criteria). Total of 2 × 24 h recalls (1st: interview & 2nd: phone) on non-consecutive days (Automated Multipass Method). Nutrient analysis with the Nutrition Data System for Research and Greek food composition tables (+newly marketed foods). FDA definition for total sugars. Lifestyle: Pre-Physical Activity Questionnaire (PAQ) and IPAQ-A Questionnaire (screen time, SES).	18.7% of children and 24.5% of adolescents had >10% of their total energy intake from added sugars. The main sources of added sugars in both age groups were sweets (29.8%) and processed/refined grains and cereals (19.1%). In adolescents, the third main contributor was sugar-sweetened beverages (20.6%). Children/adolescents with >10% of their total energy intake from added sugars were more likely to be overweight or obese compared to their peers with <10% of their total energy intake from added sugars. The predicted probability of becoming obese was also significant with higher total and added sugar consumption.
4	Karatzi et al. [4]	The Impact of Nutritional and Lifestyle Changes on Body Weight, Body Composition and Cardiometabolic Risk Factors in Children and Adolescents during the Pandemic of COVID-19: A Systematic Review	Electronic search in: PUBMED, COCHRANE, Google Scholar, and SCOPUS (up to 31 October 2021). Total of 15 studies included.	Systematic literature review	PUBMED, COCHRANE, Google Scholar, and SCOPUS RoB 2.	COVID-19 measures negatively influenced children’s and adolescents’ diets and lifestyles. Children’s and adolescents’ body weight and central fat increased. Parental presence and control resulted in better glycemic control in children with type-1 diabetes mellitus. The impact of the pandemic on the glycemic control of children with type-2 diabetes mellitus is controversial. Dietary and lifestyle changes had a differential impact on children’s hypertension prevalence.
5	Ferrer-Santos et al. [5]	Moderate-to-Vigorous Physical Activity and Body Composition in Children from the Spanish Region of Aragon	CALINA Cohort Study 308 children 7 years old (161 boys) recruited in September 2016 to September 2017.	Cross-sectional study	Assessment of PA levels (accelerometry). Body composition analysis (DXA). Questionnaires (lifestyle, perinatal factors, parental BMI, SES.	Higher percentage of boys (69.6%) met the WHO PA recommendations compared to girls (40.9%). A negative association was observed between MVPA and subtotal fat and abdominal fat. Subtotal body fat, abdominal fat, and fat mass index (FMI) were significantly lower in those classified as active. MVPA was associated with body fat.
6	Rusek et al. [6]	Changes in Children’s Body Composition and Posture during Puberty Growth	Total of 464 children/adolescents 6–16 years old (234 boys/230 girls) living in Trzebownisko Municipality, a rural area in southeastern Poland, 8 randomly selected schools (5 primary & 3 secondary).	Cross-sectional study	Tanner stage assessment of body posture (Zebris system). Body composition analysis (TANITA MC 780 MA): measurement of body weight, adiposity, lean mass, muscle mass, total body water, BMR, compartmental body composition analysis. Measurement of body height Calculation of BMI.	Pelvic obliquity was lower in older children. Age played a significant role in differences in the height of the right pelvis, and the difference in the height of the right shoulder. The content of adipose tissue (FAT%) increased with BMI and decreased with increasing weight, age, and height. FAT% was lower in boys compared to girls. Older children (puberty) had greater asymmetry in the right shoulder blade and right shoulder. Younger children (who were still prepubescent) had greater anomalies in the left trunk inclination as well as in the pelvic obliquity. Girls in puberty were characterized by greater asymmetry on the right side, including the shoulders, the scapula, and the pelvis. In boys, the problem related only to the asymmetry of the shoulder blades. Girls were characterized by a greater increase in adipose tissue and boys by muscle tissue. Significant differences appeared in children’s body posture. Greater asymmetry within the scapulas and shoulders were seen in children during puberty.
7	Hsu et al. [7]	Can Anthropometry and Body Composition Explain Physical Fitness Levels in School-Aged Children?	Total of 360 schoolchildren (180 boys/180 girls; mean age: 10 ± 0.7 years; mean BMI score 0.336). Data collected between 2013–2016 from the hospital of Chang Gung, Linkou Main Branch, Taoyuan, Taiwan.	Cross-sectional study	Questionnaires (demographics) Anthropometric measurements (body weight & height), calculation of BMI Body composition analysis Assessment of fitness level (800 m run, sit-and-reach, 1 min sit-ups, and standing long jump)	BF% was associated with the 800 m run. Sex, age, and BMI z-score were independently related to the sit-and-reach. Age, BF%, and muscle weight were associated with the 1 min sit-ups.
8	Tengku H et al. [8]	Oral Diseases and Quality of Life between Obese and Normal Weight Adolescents: A Two-Year Observational Study	Total of 397 adolescents (195 adolescents with overweight/obesity and 202 adolescents with normal weight, matched by age = 14 years at baseline and gender, were followed up for two years (2015–2017) in Malaysia).	Prospective observational cohort study	Anthropometric measurements (body weight & height), calculation of BMI and BMI weight status according to WHO criteria. Clinical examination (caries according to WHO criteria; periodontal assessment). Questionnaire (demographics, oral health related behaviors) Oral health related quality of life (OHRQoL) using the short form of the Malaysian version of Oral Health Impact Profile OHIP(M)-14.	The prevalence of dental caries was higher in the normal-weight group. Higher significant caries index was reported in the overweight/obese (OW/OB) group. The prevalence of gingivitis was high in all groups. At the 2-year follow up, the authors recorded: (1) reduction in gingival bleeding index prevalence, (2) increase in oral health-related quality of life (OHRQoL) prevalence in the OW/OB group. The findings of this study suggest that obesity status did not have an influence on the burden of oral diseases and OHRQoL.
9	Jeong et al. [9]	Positive Associations between Body Mass Index and Hematological Parameters, Including RBCs, WBCs, and Platelet Counts, in Korean Children and Adolescents	Korea National Health and Nutrition Examination Survey (KNHANES) (nationally representative survey). Children and adolescents 10–18 years old (*n* = 7997; 4259 girls and 3738 boys) recruited between 2007–2018.	Cross-sectional study	Hematological parameters (including white blood cells (WBCs), red blood cells (RBCs), hemoglobin (Hb), hematocrit (Hct), and platelets). Anthropometric measurements (body weight, height, WC), calculation of BMI. Assessment of blood pressure (SBP, DBP). Questionnaire (lifestyle, alcohol consumption, smoking, SES, previous diagnoses).	Significantly higher mean levels of WBCs, RBCs, Hb, Hct, and platelets were recorded in the obese compared to the normal weight group. BMI SDS had significant positive associations with the levels of WBCs, RBCs, Hb, Hct, and platelets. Higher BMI was associated with elevated WBC, RBC, Hb, Hct, and platelet counts in children and adolescents.
10	Hsu et al. CN (2021) [10]	Fat Mass Index Associated with Blood Pressure Abnormalities in Children with Chronic Kidney Disease	Total of 63 children and adolescents (8–18 years old) with chronic kidney disease (CKD) stage G1–G4 were recruited from the Kaohsiung Chang Gung Memorial Hospital, Taiwan, in December 2016–October 2018.	Prospective cohort study	Medical history and examination. Biochemical indices. Anthropometric measurements (body weight, height) and calculation of BMI and BMI percentiles. Body composition measurement (DEXA/calculation of fat mass index—FMI; android to gynoid fat ratio—A/G ratio). Cardiovascular assessments: 24 h ambulatory blood pressure monitoring (ABPM), arterial stiffness index, echocardiography.	Up to 63.5% of CKD children had abnormal changes in BP detected by ABPM. CKD children with abnormal ABPM were older, had higher numbers of CKD stage G2 to G4, hyperuricemia, obesity, and higher FMI z-score and A/G ratio compared to individuals with normal ABPM. Among these factors, only the FMI z-score showed an independent association with abnormal ABPM.
11	Ofenheimer et al. [11]	Using Body Composition Groups to Identify Children and Adolescents at Risk of Dyslipidemia	LEAD Cohort Study *n* = 1394 children aged 6–<18 years from the LEAD study (2011–2019) in Austria.	Cross-sectional study	Body composition analysis (DEXA). Blood samples (8 h fasting; HDL-c, LDL-c, TG + their z-scores, total cholesterol). Anthropometric measurements (body weight and height), calculation of BMI fitness level (handgrip strength). Appendicular lean mass (ALMI) = sum of the lean mass of 4 limbs (Kg) divided by height^3.5^ (m^3.5^). Fat mass index (FMI) = fat mass (Kg) divided by height^2.5^ (m^2.5^).	Different body composition groups, which are not distinguishable by BMI, exist. Children with high appendicular lean mass (ALMI) and high fat mass index (FMI) showed higher triglycerides and LDL-c, but lower HDL-c levels. Levels of these indices did not differ between those with high FMI but low (or normal) ALMI, and other body composition groups. These findings suggest that children/adolescents with high FMI who have concomitantly high ALMI should be followed closely in future studies to investigate whether they are at increased risk of cardiovascular problems.
12	Valencia-Sosa et al. [12]	Percentile Reference Values for the Neck Circumference of Mexican Children	Total of 1059 (52.9% girls) normal-weight schoolchildren aged 6–11 years from six different schools located in Acatlán de Juárez and Villa Corona, Jalisco, Mexico.	Cross-sectional study	Anthropometric measurements (body weight and height, WC, neck circumference), calculation of BMI. Body composition (skinfolds: triceps & subscapular/Slaughter equation).	Weight, height, and BMI values were higher for males (not statistically significant). The 50th percentile for females was 24.6 cm at six years old and 28.25 cm at 11 years old. The 50th percentile for males was 25.75 cm at six years old and 28.76 cm at 11 years old. Both males and females displayed a pronounced increase in neck circumference between 10 and 11 years of age. The greatest variability was found in the 11-year-old group, with an increase of 5.5 cm for males and 5.4 cm for females.
13	Thajer et al. [13]	Comparison of Bioelectrical Impedance-Based Methods on Body Composition in Young Patients with Obesity	Total of 123 children and adolescents (6–18 years) with obesity BMI: 21–59 kg/m^2^).	Cross-sectional study	BIA (TANITA and BIACORPUS) in all subjects. BOD POD and DEXA for a small patient group.	TANITA overestimated body fat percentage and fat mass relative to BIACORPUS and underestimated fat-free mass. A Bland–Altman plot indicated little agreement between methods, which produced clinically relevant differences for all three parameters. Gender-specific differences were observed with both methods, with body fat percentage being lower and fat-free mass higher in males than in females.
14	Bielec et al. [14]	Changes in Body Composition and Anthropomorphic Measurements in Children Participating in Swimming and Non-Swimming Activities	Two groups of children (11–12 years old) from Olsztyn, Poland, who attended swimming classes (*n* = 46) or participated in training activities in other sports, including, but not limited to, football, basketball, and athletics (*n* = 42).	Quasi-experimental, two-group study (non-random sampling)	Anthropometric measurements (body height and height). Calculation of BMI BIA (Tanita BC 418 MA analyzer). Students individually reported their rate of perceived exertion during training using the Pictorial Children’s Effort Rating Table (PCERT) scale.	The weekly volume of training was higher in the group of swimmers than in that playing other sports. After 12 weeks of training, body height and weight increased in both groups. However, the BMI value and adipose tissue content only increased in the group of non-swimmers. Swimmers perceived greater exertion during training than non-swimmers.
15	Trajkovic et al. [15]	Effects of After-School Volleyball Program on Body Composition in Overweight Adolescent Girls	Total of 42 overweight adolescent girls were randomly divided into a volleyball group (VG) (*n* = 22; age: 15.6 ± 0.5 years) and control group (CG) (*n* = 20; age: 15.5 ± 0.7 years).	Randomized study comparing an after-school volleyball program with traditional physical education classes in overweight female adolescents.	Description of volleyball program: Volleyball activities, played as small-sided games, two times a week after school. The experimental program lasted for 12 weeks. Volleyball was played on a court and the number of players was modified from 3 vs. 3 to 5 vs. 5 players. Duration & Context: Warm-up (10–12 min). Main part: 40 min of volleyball and 5 min cool-down. Intensity of training monitored (rate of perceived exertion) CG undertook their regular PE classes. In both groups, volleyball was excluded from regular PE classes. Assessments: - BIA (InBody Co.). - Anthropometrics (body weight, height), calculation of BMI.	A significant interaction between groups (intervention vs. control) and time (pre- vs. post) was observed for weight and BMI. Significant main effect of time was found for body fat (kg) and body fat (%). The results of the current study show that a 12-week after-school volleyball program, including 2 session/week, can improve body composition in overweight adolescent girls.
